# Right place, right time: localisation and assembly of the NLRP3 inflammasome

**DOI:** 10.12688/f1000research.18557.1

**Published:** 2019-05-17

**Authors:** Claire Hamilton, Paras K. Anand

**Affiliations:** 1 Infectious Diseases and Immunity, Department of Medicine, Imperial College London, The Commonwealth Building, Du Cane Road, London, W12 0NN, UK

**Keywords:** NLRP3, caspase-1, mitochondria, endoplasmic reticulum, Golgi, inflammasome, IL-1β, IL-18, cholesterol, SREBP2

## Abstract

The NLRP3 inflammasome is a multimeric protein complex that cleaves caspase-1 and the pro-inflammatory cytokines interleukin 1 beta (IL-1β) and IL-18. Dysregulated NLRP3 inflammasome signalling is linked to several chronic inflammatory and autoimmune conditions; thus, understanding the activation mechanisms of the NLRP3 inflammasome is essential. Studies over the past few years have implicated vital roles for distinct intracellular organelles in both the localisation and assembly of the NLRP3 inflammasome. However, conflicting reports exist. Prior to its activation, NLRP3 has been shown to be resident in the endoplasmic reticulum (ER) and cytosol, although, upon activation, the NLRP3 inflammasome has been shown to assemble in the cytosol, mitochondria, and mitochondria-associated ER membranes by different reports. Finally, very recent work has suggested that NLRP3 may be localised on or adjacent to the Golgi apparatus and that release of mediators from this organelle may contribute to inflammasome assembly. Therefore, NLRP3 may be strategically placed on or in close proximity to these subcellular compartments to both sense danger signals originating from these organelles and use the compartment as a scaffold to assemble the complex. Understanding where and when NLRP3 inflammasome assembly occurs may help identify potential targets for treatment of NLRP3-related disorders.

## Introduction

Inflammasomes are multimeric protein complexes composed of typically a Nod-like receptor (NLR) or absent in myeloma 2 (AIM2), the adaptor molecule apoptosis-associated speck-like protein containing a CARD (ASC), and the effector protease caspase-1. Upon activation, the receptor dimerises and recruits ASC via pyrin domain interactions and ASC subsequently interacts with caspase-1 via their respective CARD domains. Caspase-1 undergoes autoproteolytic cleavage to produce an active fragment which is able to cleave the precursor forms of cytokines interleukin 1 beta (IL-1β) and IL-18, leading to pro-inflammatory responses ^1– 5^. In addition to cleaving the above cytokines, caspase-1 cleaves gasdermin-D (GSDMD) to produce an active N-terminal fragment which subsequently inserts into the plasma membrane and assembles a pore, resulting in an inflammatory cell death termed pyroptosis ^6– 8^.

Of all the NLR inflammasomes characterised to date, the NLRP3 inflammasome has been the most extensively studied, largely due to its role in several infectious and inflammatory disorders ^9– 14^. Activation of the NLRP3 inflammasome is unique in that it requires two signals to assemble. The first signal involves ligation of typically a Toll-like receptor (TLR), initiating activation of the transcription factor nuclear factor kappa-light-chain-enhancer of activated B cells (NF-κB) and upregulation of pro-IL-1β and NLRP3. This initial step is often referred to as the “priming” signal. The second signal (also known as the activating signal) is induced by a wide variety of substances, including exogenous and endogenous compounds such as ATP, silica, cholesterol crystals, and alum, as well as a variety of bacterial, viral and fungal pathogens or their toxins (or both). How such a wide variety of structurally and chemically diverse entities are all able to activate the NLRP3 inflammasome has been at the forefront of inflammasome biology for the last decade. It is commonly accepted that all of these ligands do not bind NLRP3 directly and instead converge on a cellular pathway which subsequently activates NLRP3 ^15^. Mechanisms proposed for NLRP3 activation include K ^+^ efflux, mitochondrial dysfunction, production of reactive oxygen species (ROS), lysosomal rupture, and Ca ^2+^ mobilisation. Although some of these mechanisms are involved in response to many NLRP3 activators, absolute requirement of any specific mechanism with all activating signals is still to be demonstrated, thereby precluding a consensus on the central NLRP3 inflammasome-activating mechanism. A common theme among many of the proposed mechanisms is the sensing of molecules released as a result of cellular/organelle stress and dysfunction ^16– 18^. Additionally, emerging evidence has now suggested key homeostatic functions for endoplasmic reticulum (ER) and Golgi in NLRP3 activation ^18– 22^. Here, we review recent literature focusing on the roles that mitochondria, ER, and Golgi play in the localisation, assembly, and activation of the NLRP3 inflammasome. Though less studied, the requirement of these organelles in activation of other inflammasomes, when relevant, is also discussed. A critical role for lysosomes has been recognised in response to particulate ligands; however, we have not included a separate section on this organelle as it has been discussed in detail in several published reviews ^23– 25^.

## Cellular localisation of NLRP3 and ASC

Studies conducted almost a decade ago suggested NLRP3 inflammasome localisation on mitochondria and mitochondria-associated ER membranes (MAMs) ^26^. Detailed biochemical studies demonstrated that, under resting conditions, the majority of NLRP3 is located in the ER and cytosol of THP-1 cells overexpressing NLRP3. However, upon activation with NLRP3 activators monosodium urate (MSU) or nigericin, NLRP3 relocated to the perinuclear space and associated with both mitochondrial and ER markers and therefore is thought to be located on MAMs, composed of both ER and mitochondrial outer membrane fragments ^26^. A small fraction of NLRP3 was also found in the cytosol upon activation. Similarly, Yang *et al*. have demonstrated that the activated NLRP3 inflammasome is located on the mitochondria ^27^. Conversely, Wang *et al*. showed that the NLRP3 inflammasome shows no association with mitochondrial markers and was located solely in the cytosol of mouse macrophages exposed to lipopolysaccharide (LPS) and ATP ^28^. However, the location of NLRP3 may be cell-specific, as nuclear localisation was reported in CD4 ^+^ Th2 cells, whereas cytoplasmic location was observed in CD4 ^+^ Th1 cells following differentiation ^29^. In this case, nuclear NLRP3 transcriptionally regulated Th2 differentiation of CD4 ^+^ T cells but did not participate in inflammasome formation per se ^29^.

**Figure 1.**
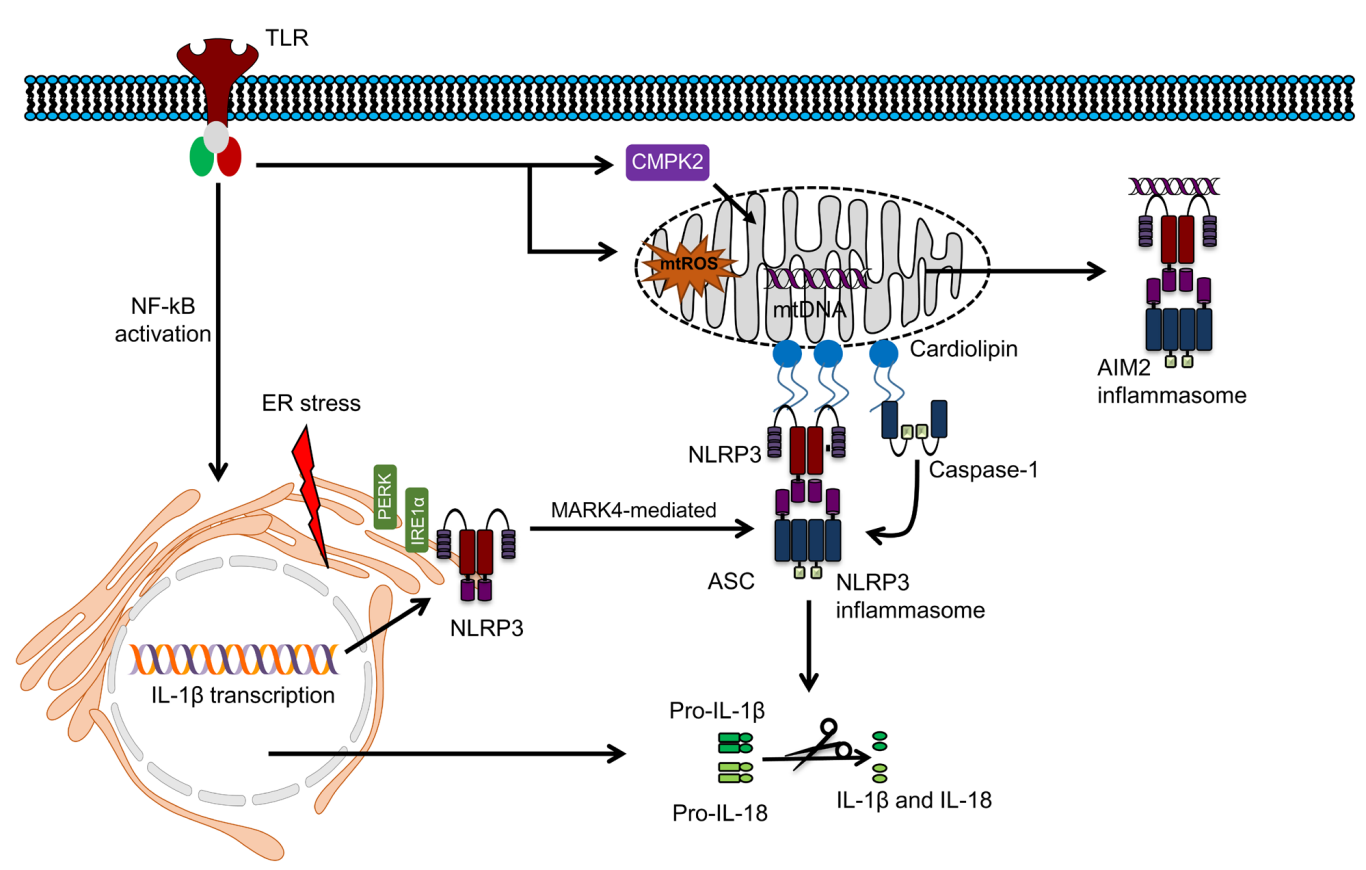
Mitochondria and ER in activation of the NLRP3 inflammasome. A proportion of NLRP3 has been shown to reside in the ER prior to activation and translocate to the mitochondria or mitochondria-associated membranes during activation. The translocation of NLRP3 to the mitochondria has been shown to be dependent on microtubule rearrangement and microtubule kinase MARK4. Activation of the NLRP3 inflammasome requires two signals. The first signal, referred to as NLRP3 “priming”, requires activation of NF-κB (for example, through TLR stimulation) and the upregulation of NLRP3 and IL-1β. The second activating signal has been shown to be mediated via several mechanisms. TLR priming induced upregulation of mtDNA via CMPK2, and the upregulation of mtROS has been shown to activate the NLRP3 at the mitochondria. mtDNA may also be able to activate the AIM2 inflammasome. ER stress has also been implicated in NLRP3 inflammasome activation, and ER stress–induced activation of the UPR response pathway, including UPR sensors IRE1α and PERK, is required for NLRP3 inflammasome activation. Following activation, the NLRP3 inflammasome leads to the activation of caspase-1 and subsequent cleavage of IL-1β and IL-18. AIM2, absent in myeloma 2; ASC, apoptosis-associated speck-like protein containing a CARD; CMPK2, cytidine/uridine monophosphate kinase 2; ER, endoplasmic reticulum; IL-1β, interleukin-1 beta; IL-18, interleukin-18; IRE1α, inositol-requiring protein 1 alpha; MARK4, microtubule-affinity regulating kinase 4; mtDNA, mitochondrial DNA; mtROS, mitochondrial reactive oxygen species; NF-κB, nuclear factor kappa-light-chain-enhancer of activated B cells; PERK, protein kinase R-like endoplasmic reticulum kinase; TLR, Toll-like receptor; UPR, unfolded protein response.

The recruitment of NLRP3 to MAMs may be dependent on mitochondrial cardiolipin or the presence of mitochondrial antiviral signalling (MAVS), an outer mitochondrial membrane protein involved in retinoic acid–inducible gene I (RIG-I)-mediated interferon (IFN) responses ^30– 32^. Additionally, the translocation of NLRP3 to mitochondria may rely on the presence of a short sequence in the N-terminal domain of human NLRP3 ^30^; although murine NLRP3 differs in this minimal N-terminal sequence, it too was found to be associated with mitochondria upon activation ^32^. Other studies found a role for microtubules in the transport of NLRP3 to mitochondria and this was aided by microtubule-affinity regulating kinase 4 (MARK4), which helped position NLRP3 to mitochondria for effective “speck” formation and optimal inflammasome activity ( Figure 1). mCherry-tagged NLRP3 was shown to move along microtubules towards mitochondria in nigericin-activated THP-1 cells, a response that was diminished in MARK4 knock-down cells ^33^. In agreement, inhibition of tubulin polymerisation by either colchicine or nocodazole diminished NLRP3 inflammasome activation ^33, 34^. The localisation of NLRP3 on or in close vicinity to mitochondria offers an obvious advantage to the cell as any disturbance in cellular homeostasis leading to mitochondrial dysfunction would result in efficient sensing and activation of NLRP3. Studies on the localisation of ASC within the cell are somewhat contradictory; Misawa *et al*. demonstrated the localisation in the mitochondria, cytosol, and nucleus under resting conditions in primary bone marrow–derived macrophages (BMDMs) ^34^. Once an activating signal is sensed, a dynein-dependent mechanism transports ASC on the mitochondria in close proximity to NLRP3 on the ER ^34^. This transport was shown to be independent of K ^+^ efflux and mitochondrial ROS (mtROS). Additionally, other reports have shown ASC to reside exclusively in the cytosol and in the nucleus under resting conditions and to associate with mitochondria upon NLRP3 inflammasome activation ^26, 35^. Whether the nature of the upstream stimuli calibrates the localisation of the NLRP3 inflammasome to distinct subcellular compartments is still unclear, as is the localisation of other inflammasome-forming receptors.

## Mitochondria

### Mitochondrial dysfunction leads to NLRP3 inflammasome activation

Positioning of NLRP3 following activation on MAMs/mitochondria is thought to enable immediate recognition of mitochondrial damage, which has been proposed as a central event downstream of various NLRP3-activating stimuli. These damage signals include mtROS, mitochondrial DNA (mtDNA) and cardiolipin ( Figure 1) ^26, 36, 37^.

Cellular ROS was initially shown to activate the NLRP3 inflammasome in an NAPDH-dependent manner; however, further studies showed that ROS generated by the mitochondria is sufficient to lead to NLRP3 activation ^26, 38, 39^. Specific inhibition of mtROS significantly diminishes NLRP3 inflammasome activation ^40^. Yu *et al*. demonstrated that mitochondrial damage following NLRP3 inflammasome activation occurs in a caspase-1–dependent manner, resulting in the release of mtROS ^41^. Caspase-1 was demonstrated to amplify this response by inhibiting mitophagy, the process of removal of damaged mitochondria, which then contributed to pyroptosis induction ^41^. Conversely, ROS production has been shown to be dispensable for NLRP3 inflammasome activation, and the significance of K ^+^ efflux has been highlighted ^42^, thereby suggesting these as independent NLRP3 activation mechanisms. How increases in mtROS production activate NLRP3 is still unclear, although TXNIP has been implicated by various studies ^43– 45^. TXNIP is a negative regulator of the antioxidant thioredoxin (TRX) and is thought to dissociate from TRX in the presence of ROS, allowing TXNIP to bind to NLRP3. In agreement, NLRP3 inflammasome activation is impaired in TXNIP ^-/-^ mice ^46^. The precise mechanism remains unresolved. Furthermore, mutations in mtDNA-encoded cytochrome b gene in patients with fibromyalgia are associated with mitochondrial dysfunction and increased ROS production, leading to enhanced caspase-1 cleavage, and IL-1β and IL-18 secretion in fibroblasts and serum respectively ^47^. Increases in ROS have previously been implicated in other inflammatory disorders ^48, 49^.

In addition to mtROS, the release of mtDNA—in particular, oxidised mtDNA—in response to mitochondrial dysfunction activates the NLRP3 inflammasome ^36, 50, 51^. NLRP3 inflammasome priming via TLRs has been shown to induce mtROS production and, more recently, the production of mtDNA ^52, 53^. The latter is dependent on MyD88/TRIF- and IRF-1-mediated upregulation of mitochondrial deoxyribonucleotide kinase, CMPK2, an enzyme with rate-limiting activity in mtDNA synthesis ^53^. The newly synthesised mtDNA (made during priming) was found to be necessary to generate oxidised mtDNA fragments when cells were subsequently exposed to an NLRP3-activating signal ( Figure 1) ^53^. Of note, oxidised DNA from any cellular source can activate the NLRP3 inflammasome. In fact, mtDNA contributes to inflammation in diseases such as atherosclerosis and inflammatory kidney disorders, and the inhibition of its release by exposure to agents that maintain mitochondrial integrity prevents NLRP3 inflammasome activation ^54– 59^. Contrary to the above, TLR-dependent priming also restricts inflammasome activation through efficient removal of damaged mitochondria. TLR-activated NF-κB pathway induces prolonged accumulation of the autophagic receptor p62/SQSTM1, which targets damaged mitochondria for clearance by mitophagy ^60^, thereby preventing NLRP3 over-activation ^61^. Whether inhibition of mtDNA release or maintenance of mitochondrial integrity (or both) will translate into novel therapeutics for disorders in which NLRP3 inflammasome is dysregulated is yet to be seen.

A small number of studies have implicated mtDNA in the activation of other inflammasome complexes. Mitochondrial damage by *Pseudomonas aeruginosa* leads to the release of ROS and mtDNA; the latter was shown to bind and activate the NLRC4 inflammasome. In agreement, abolition of mtDNA by DNase I treatment resulted in reduced caspase-1 activation and IL-1β secretion following *P. aeruginosa* infection ^62^. However, caspase-1 activation was not completely abolished by DNase I in *Nlrc4* ^−/−^ macrophages, suggesting roles for other inflammasomes, and the authors attributed this to AIM2 inflammasome activation. Although NLRP3 is not thought to be involved during *P. aeruginosa* infection, a role for NLRP3 was not experimentally ruled out ^62, 63^. Dang *et al*. also demonstrated that mtDNA, released as a result of loss of mitochondrial integrity by elevated cellular cholesterol levels, can activate the AIM2 inflammasome in macrophages lacking cholesterol-25-hydroxylase. In wild-type cells, production of 25-hydroxycholesterol (25-HC) by cholesterol-25-hydroxylase repressed cholesterol biosynthesis, thereby maintaining mitochondrial integrity ^64^. However, only a small redundant role for NLRP3 was found in the above study, although mtDNA is known to activate this inflammasome ^36^. As the AIM2 inflammasome can recognise both foreign and self-DNA, it is likely that it can also recognise mtDNA; however, further studies are required to confirm these findings. Whether mtDNA has the ability to directly activate multiple inflammasomes ^65^ or whether upstream stimuli direct differential activation of NLRP3 and AIM2 inflammasomes requires further clarification.

### NLRP3 is recruited to and binds mitochondrial cardiolipin

Cardiolipin, a phospholipid localised to the inner mitochondrial membrane (IMM), has also been linked to NLRP3 inflammasome activation. Upon NLRP3 activation or in the presence of stimuli that destabilise mitochondria, cardiolipin redistributes to the outer leaflet of the mitochondrial membrane (OMM), where NLRP3 anchors through its leucine-rich repeat (LRR) domain ^37, 66^ ( Figure 1). Depletion of cardiolipin or abrogation of cardiolipin synthesis by culturing cells in the presence of palmitate (C16:0), a saturated long-chain fatty acid, blunted inflammasome activation in J774A.1 macrophages ^37^. Similarly, small interfering RNA (siRNA) knock-down of cardiolipin synthase reduced NLRP3 activation ^37^. Corroborating these findings, activation of the NLRP3 inflammasome, by HIV reverse transcriptase inhibitor abacavir, was abrogated following inhibition of cardiolipin synthase-1 ^37^. Furthermore, cardiolipin-dependent NLRP3 inflammasome activation by the antibiotic linezolid, which induces mitochondrial toxicity, was ROS-independent ^37^. Thus, mitochondrial dysfunction, which induces ROS under certain conditions, and not ROS itself, may be responsible for NLRP3 inflammasome activation. Additionally, recent work from the group established that NLRP3 is recruited to the mitochondria at the priming stage and binds to cardiolipin on the OMM ^67^. Interestingly, the movement of cardiolipin to the OMM was found to be dependent on ROS produced during the NLRP3 priming step. Surprisingly, caspase-1 also binds cardiolipin at the mitochondria, while ASC was recruited only following NLRP3 activation ^67^. The binding of both caspase-1 and NLRP3 to mitochondrial cardiolipin validates mitochondria as a scaffold for inflammasome activation, thereby amplifying its activation by bringing molecules together ( Figure 1). In line with this, other immune signalling pathways initiated at the mitochondria, including the MAVS complex, occur in a similar manner ^68^. Additionally, these studies identify a role for priming beyond the upregulation of NLRP3 and pro-IL-1β.

## Endoplasmic reticulum

### Endoplasmic reticulum stress triggers caspase-1 activation

NLRP3 has been shown to reside in the ER prior to its activation, and ER dysfunction has been reported to trigger inflammasome activation ( Figure 1). The ER is a membrane-bound organelle that is critical for protein folding, assembly and modification in addition to being the site for lipid synthesis and Ca ^2+^ homeostasis. Cellular stress leading to accumulation of misfolded or unfolded proteins induces an unfolded protein response (UPR) aimed at restoring ER homeostasis by regulating levels of transcription, translation, and protein folding ^69^. The UPR response activates several stress sensors located in the ER, including inositol-requiring protein 1 alpha (IRE1α), protein kinase R-like endoplasmic reticulum kinase (PERK), and activating transcription factor 6 (ATF6). These sensors subsequently regulate downstream cytosolic effectors and signalling pathways, including proteasomal degradation, autophagy, and antioxidant defence mechanisms. Prolonged ER stress induces an inflammatory response and cell damage, thereby triggering NLRP3 inflammasome activation ^18, 70– 73^. An initial study suggested that ER stress activated the NLRP3 inflammasome via a UPR-independent pathway, as short hairpin RNA (shRNA) knock-down of either *Ire1α* or *Perk* or macrophages obtained from *Atf6α* ^−/−^ mice released comparable IL-1β following NLRP3 activation ^74^. However, the authors also noted that not all NLRP3 activators induced ER stress, indicating that this may be just one mechanism by which the NLRP3 inflammasome is activated. In agreement, Bronner *et al*. found that ER stress and IRE1α were not induced in mouse BMDMs with the canonical NLRP3 activator ATP, although they were induced during *Brucella abortus* infection ^18^. During *B. abortus* infection, IRE1α-initiated mtROS production recruited NLRP3 to mitochondria. Mechanistically, IRE1α activated TXNIP and caspase-2, leading to truncation and activation of the mitochondrial protein Bid, which resulted in mitochondrial damage and release of mitochondrial damage-associated molecular patterns (DAMPs) that promoted NLRP3 activation ^18^. Surprisingly, NLRP3 was required to activate caspase-2/Bid upstream of mitochondrial damage, suggesting a role for NLRP3 in initiating mitochondrial damage by a feed-forward loop. IRE1α and its activation of TXNIP have been implicated in NLRP3 inflammasome activation in other studies ^19, 75– 78^. Similarly, inhibition of the ER stress sensor PERK was shown to reduce caspase-1 activation and IL-1β secretion in J744.1 macrophages, although how PERK inhibition decreases NLRP3 activation was not determined ^79^. Targeting IRE1α to dampen ER stress–induced NLRP3 inflammasome activation has shown benefits in a wide variety of inflammatory conditions ^75, 76, 80– 85^. These studies again suggest mitochondrial damage as the downstream mechanism by which ER stress initiates NLRP3 inflammasome formation. Additionally, this work may indicate that the ER is the site where NLRP3 activation is initiated before the inflammasome is assembled in the cytosol or at the mitochondria/MAMs.

In addition to being implicated in the activation of the NLRP3 inflammasome, ER stress is thought to play a role in the activation of the NLRP1 inflammasome. NLRP1 expression in HeLa cells is upregulated upon induction of ER stress by tunicamycin and thapsigargin, which inhibit the N-linked glycosylation of proteins and sarcoplasmic/endoplasmic reticulum Ca ^2+^-ATPase (SERCA) respectively. Upregulation of *Nlrp1* involved IRE1α and PERK, and siRNA knock-down of either *Ire1α* or *Perk* abrogated increase in NLRP1 expression ^86^. Consistent with this, studies have shown a link between ER stress and NLRP1 upregulation in leukaemia and cardiovascular injury models ^87, 88^. Thus, as suggested by studies discussed below, ER seems to be a key subcellular site to regulate inflammasome activation.

### Endoplasmic reticulum calcium homeostasis in inflammasome activation

The ER is also the site at which Ca ^2+^ homeostasis occurs, and Ca ^2+^ mobilisation has been implicated in NLRP3 inflammasome activation. Blockade of the ER-resident calcium channel IP3R led to reduced NLRP3 inflammasome activation in mouse macrophages ^89, 90^. Other studies have challenged these claims, showing no role for Ca ^2+^ and indicating that K ^+^ efflux is more important ^42, 91^. It can be expected that, as with other mechanisms of NLRP3 activation, Ca ^2+^ mobilisation from the ER activates the NLRP3 inflammasomes only under specific conditions. Similarly, it is possible that Ca ^2+^ mobilisation precedes ER stress or is a consequence of ER dysfunction and therefore happens to occur alongside ER stress–induced NLRP3 activation.

### Endoplasmic reticulum cholesterol levels regulate NLRP3 activation

The ER not only is involved in lipid synthesis but also is the site at which cholesterol levels within the cell are sensed and regulated. Cellular cholesterol homeostasis is achieved by maintaining an equilibrium between *de novo* synthesis at the ER, exogenous cholesterol uptake in the form of low-density lipoprotein (LDL), and cholesterol efflux programs. LDL is endocytosed by LDL receptor and subsequently trafficked through the endosomal system to other subcellular compartments, including the ER, plasma membrane, and Golgi. Blockade of cholesterol efflux from the lysosome was recently demonstrated to inhibit NLRP3 inflammasome activation in mouse macrophages, an effect attributed to decreased cholesterol within the ER ^21^. Similarly, inhibition of cholesterol biosynthesis in the ER by exposing cells grown in lipoprotein-deficient media to statins dampened NLRP3 inflammasome activation ^21^. Additionally, the ER-localised cholesterol-sensing transcription factor sterol regulatory element-binding protein 2 (SREBP2) has been implicated in NLRP3 inflammasome activation ^92^. The cholesterol metabolite, 25-HC, has also been shown to activate the NLRP3 inflammasome by induction of K ^+^ efflux, mtROS production, and activation of the cholesterol transcriptional regulator liver X receptors (LXRs) ^93^. Thus, either cholesterol may be involved in directly influencing inflammasome complex formation or ER cholesterol content may support NLRP3 in achieving the necessary conformation. Regardless, these studies emphasise a critical role for ER in inflammasome activation.

## The Golgi complex

### NLRP3 translocation near the Golgi is required for inflammasome activation

**Figure 2.**
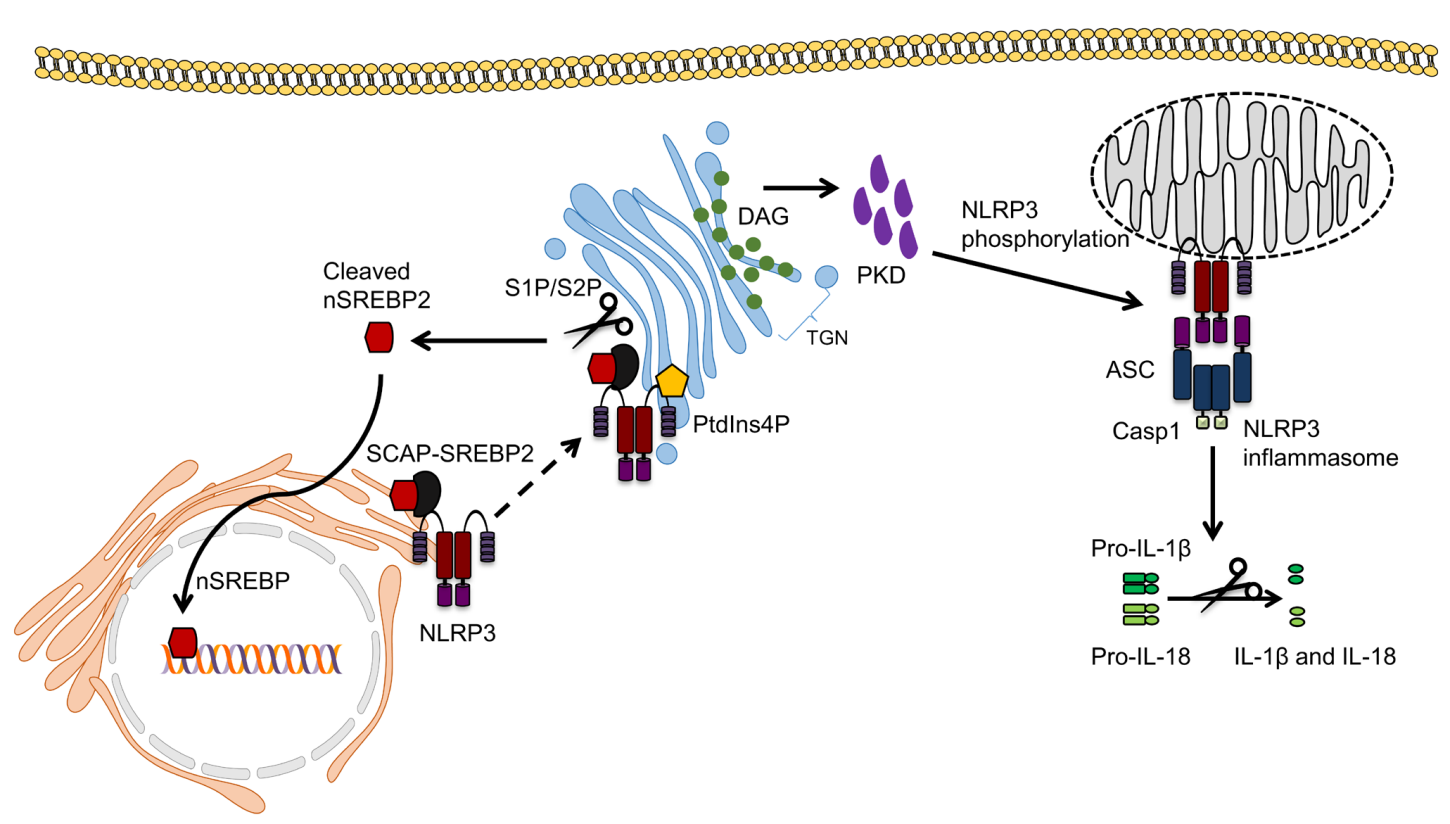
Emerging role of the Golgi apparatus in NLRP3 inflammasome activation. NLRP3 localised at the endoplasmic reticulum has been shown to translocate to the Golgi via binding to the SCAP-SREBP2 complex, which regulates cholesterol homeostasis. SREBP2 is cleaved at the Golgi by S1P and S2P to yield the active nuclear form of SREBP2, which then translocates to the nucleus and upregulates genes involved in cholesterol synthesis. NLRP3 has also been shown to be recruited to the TGN through ionic bonds formed with PtdIns4P. The Golgi then acts as a scaffold for NLRP3 assembly. Finally, DAG accumulation at the Golgi recruits and activates PKD, which is required for NLRP3 phosphorylation at the mitochondria, leading to its release and activation at mitochondrial-associated membranes. ASC, apoptosis-associated speck-like protein containing a CARD; DAG, diacylglycerol; IL-1β, interleukin-1 beta; IL-18, interleukin-18; PKD, protein kinase D; PtdIns4p, phosphatidylinositol-4-phosphate; S1P, site-1 protease; S2P, site-2 protease; SREBP2, sterol regulatory element-binding protein 2; TGN, *trans*-Golgi network.

Although roles for mitochondria and ER have been suggested by many labs in the last decade, a few recent studies have also implicated Golgi in inflammasome activation. The Golgi apparatus is important for the modification and transport of proteins and lipids within the cell. Zhang *et al*. first implicated the Golgi in NLRP3 inflammasome activation. Following treatment with NLRP3 activators, the levels of diacyl glycerol (DAG) at the Golgi increased and this coincided with the localisation of NLRP3 on MAMs adjacent to the Golgi ^20^. Disruption of Golgi integrity with brefeldin A reduced caspase-1 activation, IL-1β secretion and ASC speck formation following NLRP3 inflammasome activation, a result which was corroborated in a subsequent study ^94^. This effect was attributed to protein kinase D (PKD), a DAG effector, which phosphorylated NLRP3 at a conserved residue (Ser293) in its NBD domain, allowing its release from MAMs to form an inflammasome complex within the cytosol ( Figure 2). Of note, PKD phosphorylated after NLRP3 self-oligomerised and PKD inactivation both retained self-oligomerised NLRP3 at MAMs and reduced NLRP3 inflammasome activation. Interestingly, PKD inhibition in cells from patients with cryopyrin-associated periodic syndrome (who exhibit spontaneous NLRP3 oligomerisation) also showed reduced NLRP3 activation ^20^. This added to previous studies that indicate that post-translational modifications can both positively and negatively affect NLRP3 activation ^95, 96^. Furthermore, this study highlights cross-talk between Golgi and MAMs during NLRP3 inflammasome activation by means of DAG accumulation. In support of this, a recent study demonstrated recruitment of NLRP3 to the *trans*-Golgi network (TGN) through ionic bonding between NLRP3 and phosphatidylinositol-4-phosphate (PtdIns4P) in the membrane of the Golgi ( Figure 2) ^22^. The authors found that the TGN dispersed and formed vesicles in the perinuclear space that co-localised with overexpressed NLRP3 following inflammasome activation in HeLa cells. Similar results were shown in primary BMDMs lacking ASC; therefore, whether the full assembly of the inflammasome complex occurs at the TGN was not addressed. However, in that study, in contrast to previous studies, NLRP3 was not found to be associated with mitochondria, and instead the TGN, rather than the mitochondria, provided the scaffold for NLRP3 inflammasome activation ^22^. Furthermore, translocation of NLRP3 from the ER to the Golgi, this time requiring SREBP and SREBP cleavage-activating protein (SCAP), has also been reported in primary mouse macrophages ^92^. When cholesterol levels within the cell are low, the SCAP-SREBP2 complex translocates from the ER to the Golgi, where SREBP2 is cleaved into its active form by site-1 protease (S1P) and site-2 protease (S2P), and translocates to the nucleus to transcribe genes involved in cholesterol biosynthesis and uptake ^97^. The latter study demonstrated that NLRP3 partially co-localises with SCAP-SREBP2 and translocates to the Golgi, and that inhibition of SCAP-SREBP2 translocation abrogated NLRP3 inflammasome activation ( Figure 2). Subcellular fractionation studies showed NLRP3 in the Golgi as well as in the mitochondrial fraction with SCAP. This correlates with findings from Zhang *et al*., who suggested that NLRP3 activation occurs in close proximity to both the Golgi and the mitochondria ^20^. Whether multiple mechanisms can independently initiate NLRP3 recruitment to the Golgi requires further work and so does the role of NLRP3 phosphorylation in inflammasome activation.

## Concluding remarks

The intracellular milieu of the cell is vital for maintenance of cell function as well as the many cellular processes. Damage to organelles, caused by either endogenous agents or invading pathogens, has been demonstrated to activate NLRP3 inflammasome, leading to pro-inflammatory cytokine release and cell death via pyroptosis. Although a consensus has yet to be reached as to the precise location of the resting NLRP3 and the inflammasome complex, several studies have indicated that NLRP3 translocates to the mitochondria or MAMs following activation ( Table 1). The mitochondria are integral to providing the cell with its source of energy; therefore, it is conceivable that any insult to this compartment is sufficient to be perceived as a danger signal. NLRP3 localisation on or in proximity to mitochondria upon activation supports the notion that it is competently positioned to sense danger signals originating from this organelle. This is backed by the numerous studies demonstrating an association between loss of mitochondrial integrity and NLRP3 inflammasome activation. The direct roles of other subcellular compartments in inflammasome assembly remain to be established, although studies have shown vital roles for ER stress signalling and ER cholesterol content in NLRP3 inflammasome activation. Unravelling the input of distinct organelles in NLRP3 inflammasome assembly will provide crucial insights into how cells sense and coordinate the activation of the NLRP3 inflammasome. This new understanding would enable the development of therapeutics that calibrate NLRP3 activation to maintain cellular homeostasis.

**Table 1.** Localisation of NLRP3 before and after NLRP3 inflammasome activation.

	Cells and method employed	Resting NLRP3	Activated NLRP3/NLRP3 inflammasome	Reference
1.	THP-1 cells overexpressing FLAG-tagged NLRP3 and ASC Subcellular fractionation and confocal and electron microscopy	ER and cytosol	MAMs	Zhou *et al*. ^26^ (2011)
2.	Peritoneal macrophages from C57BL/6 mice Immunofluorescence microscopy	-	Cytosol	Wang *et al*. ^28^ (2013)
3.	HEK-293T overexpressing NLRP3 – Confocal microscopy Wild-type and *Asc ^−/−^* immortalised BMDMs – Subcellular fractionation	Cytosol	Mitochondria	Subramanian *et al*. ^30^ (2013)
4.	BMDMs – Immunofluorescence microscopy	ER	Mitochondria/ER (microtubules bring ASC on mitochondria and NLRP3 on ER together)	Misawa *et al*. ^34^ (2013)
5.	BMDMs – Subcellular fractionation and immunofluorescence microscopy	-	Mitochondria	Yang *et al*. ^27^ (2015)
6.	BMDMs and THP-1 cells – Confocal microscopy	Cytosol	MAMs → cytosol	Zhang *et al*. ^20^ (2017)
7.	HeLa cells overexpressing NLRP3 – Subcellular fractionation and fluorescence microscopy *Asc ^−/−^* BMDMs – Immunofluorescence microscopy	Cytosol and *trans*- Golgi network	*Trans*-Golgi network	Chen and Chen ^22^ (2018)

ASC, apoptosis-associated speck-like protein containing a CARD; BMDM, bone marrow–derived macrophage; ER, endoplasmic reticulum; MAM, mitochondria-associated endoplasmic reticulum membrane.
